# Review of the impact of urban parks and green spaces on residence prices in the environmental health context

**DOI:** 10.3389/fpubh.2022.993801

**Published:** 2022-09-07

**Authors:** Kaida Chen, Huimin Lin, Shuying You, Yan Han

**Affiliations:** ^1^College of Landscape Architecture and Art, Fujian Agriculture and Forestry University, Fuzhou, China; ^2^Department of Urban Planning, National Cheng Kung University, Tainan, China; ^3^International Digital Economy College, Minjiang University, Fuzhou, China; ^4^Department of Spatial Culture Design, Kookmin University, Seoul, South Korea

**Keywords:** urban parks, green spaces, residence price, environmental health, healthy city

## Abstract

Urban parks have consistently played an important role in people's living environment, reflecting house prices and the extent of the people's attention. Although many studies have been conducted in this filed, the consolidated related research has not been discussed often. Therefore, related papers on the impact of urban park green spaces on housing prices in recent years should be sorted out. Different choices of urban parks and green areas will undoubtedly influence research methods, housing preferences and the nature of the effects. Consequently, a logical framework of previous studies must be constructed. This study will review the literature from four aspects: (i) review of research methods on how park green spaces affect home prices (i.e., Research techniques, such as hedonic price analysis methods, geographically weighted regression models and neural network models, are frequently used in studies, and methodological advancements have helped the field advance); (ii) examining the varying effects of the same or similar types of parkland on home values; (iii) review of studies on the subject, analyzing variations in the scope and degree of the effects of various parks on home values in terms of such factors as park size, accessibility and serving size and (iv) review of innovative research perspectives, translating the issue of impact of parklands on housing prices into a study of the capitalization and amenity of parklands.

## Introduction

People are increasingly demanding high-quality living environments as modern civilization spreads globally. The physical environment is an essential determinant of environmental health (1–3), and parklands are amongst the most significant measures of the quality and wellness of the urban environment. Particularly, parks are public green spaces in cities, serving as places for recreation, ecological maintenance, environmental beautification and disaster mitigation and refuge, apart from providing a variety of recreational amenities and services Park green spaces are directly and indirectly beneficial to city dwellers (4) and have the advantages of enhancing aesthetics and physical and mental health, regulating microclimate to reduce energy consumption and providing habitat for wildlife, as well as carbon sequestration (5). Parklands have become amongst the most important considerations of people when buying a property, resulting from the focus on how living near parklands benefits wellbeing and emotional health. Thus, effects of parks on people's living environments is increasingly becoming significant.

Living standards are substantially influenced by environmental health, and the costs (property prices) of high-quality homes reflects this idea directly. Banzhaf and Farooque (6) noted that housing prices are a significant indicator of how people live. As such, they may be used as a criterion to assess a location's livability or attractiveness (7, 8). This idea is particularly relevant when examining the impact of urban environmental health concerns on the assessment of living standards. Given the likelihood that park planning would directly affect people's decisions to buy homes, several academics have begun to investigate the effects of urban parklands on real estate prices. Accordingly, assessing the external economic value of parklands to measure their impact on residential prices provides insights into people's preferences when choosing where to live and also provides important advice for urban planning and real estate development decisions (9). Hence, the impact of urban parklands on prices of residential spaces has become of considerable research interest.

Although the majority of prior research has supported the notion that urban parks may increase the value of residential spaces by enhancing residential amenities, studies and surveys have revealed that parks can, in some circumstances, devalue real estate. For example, unkempt or abandoned amenities, intrusive noise and lights and lack of parking spots on busy streets can have detrimental effects on the value of real estate near parks (10). Accordingly, reviewing the extensive and intricate studies conducted on the issue is evidently important and fascinating.

The three modeling techniques that have been most often employed in this type of study are the hedonic pricing, geographically weighted regression and neural network models (11, 12). The majority of research on how urban parks affect home prices has relied on hedonic price models in conjunction with recent methods, such as geographically weighted regression models, which measure how much buyers are willing to pay for parklands surrounding their homes and use price models to analyze the relationship between parklands and home prices. Synthetic neural network models have become a significant advancement in this field of forecasting in recent years. The reason is that these models are more accurate than conventional hedonic pricing models at anticipating the effects of parks on home values.

Prior research has demonstrated that the effects of various types of parks on home prices vary. Therefore, different parklands could have a certain amount of appreciation and depreciation on their impact on neighborhood house prices by considering the extent to which different sizes, service radii and accessibility of parklands have an impact on home prices. Owing to differences in how purchasers perceive the demand for parks, different park types' effects on house prices can vary significantly between regions and countries. Additionally, different properties are affected differently by the same sort of park. An analysis of the impact of parklands on house prices in terms of market segmentation effects shows heterogeneity in the impact of the same type of park on house prices over time, space and property.

Meanwhile, there are many other different viewpoints on the effects of urban parklands on property prices. Accordingly, some academics have proposed that the research strategy be expanded to include studies on such topics as capitalization of parkland issues and issue of amenity. Such a novel point of view gives government funding for parks and urban green space development a markedly intuitive reference meaning, thereby helping encourage equalization of park green space services and enhancing people's wellbeing.

**Figure 1:**
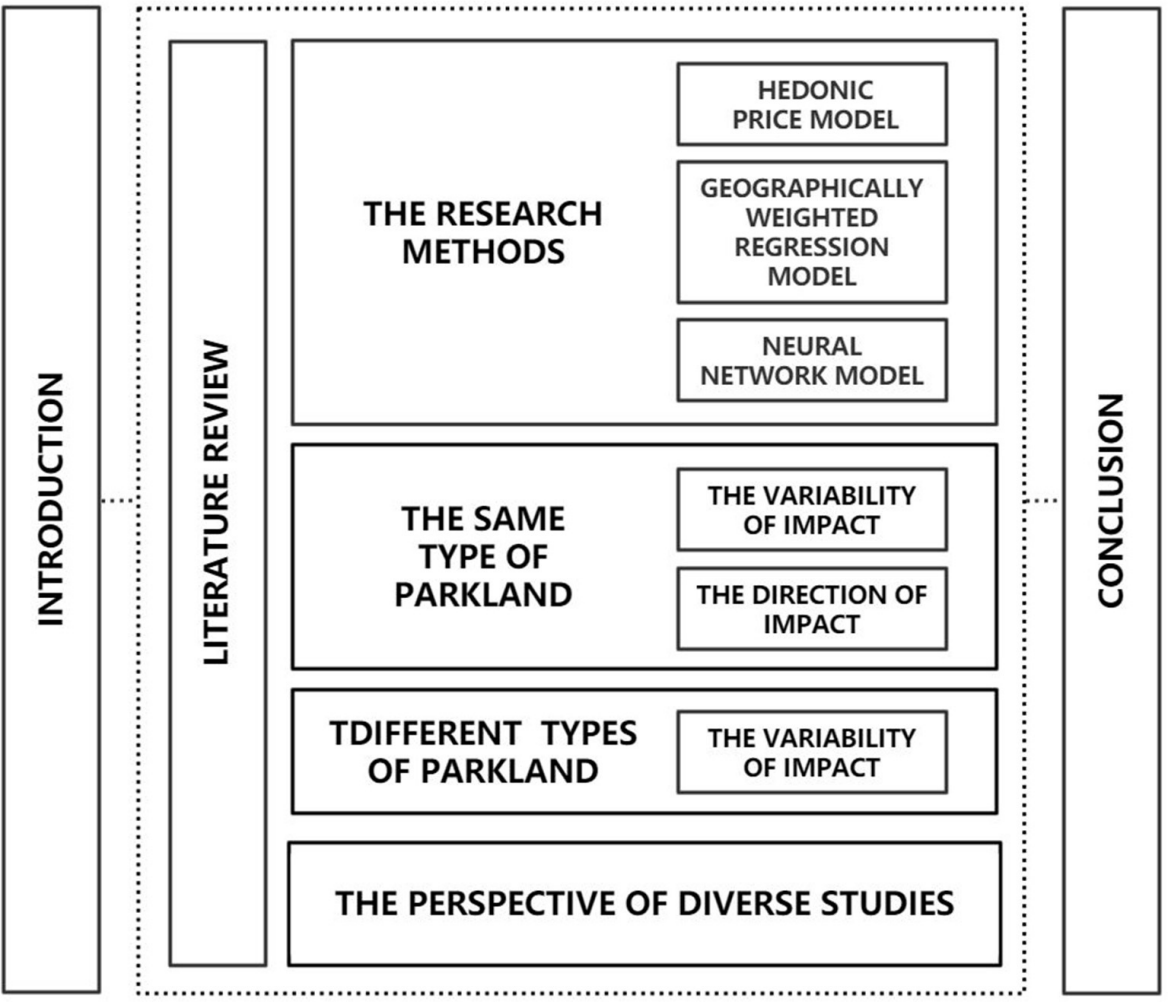
Frame diagram.

To provide theoretical and literature support for future research on the influence of park green spaces on residential prices, this article will build on the body of existing research on the role of urban park green spaces in affecting residential prices, thoroughly classify the pertinent studies and construct a logical and clear literature framework. This article is divided into three main sections (Figure 1): background introduction, followed by a classification of the literature review and a conclusion of the review.

## Literature review

The development and planning of parklands have a considerable impact on urban real estate prices, apart from enhancing and enriching the lives of the people who live there. The review, which was conducted in the following order and examined the literature in terms of research techniques, research viewpoints, comparative contrasts and reviews of significance, presents the following aspects: (i) review of research methods on how parklands affect home prices; (ii) overview of variability on the impact of the same or the same type of parkland on house prices; (iii) overview of how different types of parkland affect home prices differently and (iv) overview of other varied research perspectives.

### Theme I: Review of research methods on the impact of parklands on house prices

The hedonic price approach has a strong analytical function in terms of spatial relationships and is widely used in the study of natural green space because it can explore micro-level performance. Lancaster (13) suggested that the hedonic price model is a well-established method based on consumer theory. To analyze the effects of parklands on house values, typical components in real estate price fluctuations should be broken down by quantifying the amount that buyers are ready to pay for green spaces around their homes. Earnhart (14) used a joint analysis of Hedonic pricing to precisely quantify the economic and aesthetically pleasing benefits connected to the availability and caliber of environmental facilities in residential communities. Altunkasa and Uslu (15) demonstrated the spatial viability of parklands in the housing market through a pricing assessment approach to gather systematic data that may affect physical planning decisions for future urban development zones. Numerous studies have been conducted in this area on the use of non-market valuation methodologies, particularly the hedonic pricing approach (also known as the hedonic model) to calculate the value of urban parks and greenery to inform urban greening policy. Melichar and Kaprová (16) used a Hedonic price approach as basis in utilizing data from the real market in Prague, the largest urbanized region in the Czech Republic, and discussing the impact of several services provided by the urban environment on house prices in urban areas. Lee et al. (17) used a hedonic price model to study the Busan region chronologically over the period 1960–2011 for house sales and price data. They concluded that the larger the apartment building and the further away from the underground station and park, the lower the house price. Chen (18) explained that urban parks and green spaces are equivalent to such services as transportation and hospitals, and their direct benefits cannot be calculated based on home values. Lai (19) assessed the ecological benefits of parks and green spaces in Kaohsiung City using a typical price model and GIS spatial analytic methodologies. Their results revealed that the closer to parks and green spaces, the higher the property prices.

To deal with the geographical and temporal non-stationarity in real estate market data, researchers have included the spatio-temporal impact of house price movements into geographically weighted regression (GWR) models. This technique has allowed for a better fit to the findings of studies in areas relevant to house prices. Tang et al. (20) evaluated two models to show the spatial heterogeneity of property prices in Shanghai communities and the effects of various influencing factors: regionally weighted regression model and global ordinary least squares (OLS) technique. They concluded that GWR performs better than the global parameter estimates offered by OLS in breaking down local parameters. Moreover, they indicated that GWR can offer insights into the complex relationship between house prices and spatial influencing factors by presenting an overall picture of urban house prices. Zhang et al. (21) used spatial GWR as basis in investigating and assessing the factors impacting the spatial variance of housing in Harbin City. Their findings indicated that the primary factors impacting spatial disparities in home prices are schools, hospitals and greening characteristics. Parks and subways had less of an effect on home prices, whereas commercial centers and building age could raise home values.

In non-linear multivariate forecasting, synthetic neural network models that have recently gained popularity have excelled and significantly improved forecasting of home prices. House prices and property values are typically estimated using the hedonic price model. This model is straightforward, simple to use and can be utilized to determine the relationship between various variables and home prices. However, traditional hedonic price models are primarily linear and logarithmic models, and both have limitations. The linear model is less effective in explaining the state of multiple non-linear variables, whilst the logarithmic model is more vulnerable to extreme values in the data set and has accuracy issues. Xu et al. (22) used the error sum of the minimum standard absolute value of the linear combination model to calculate the weight of each model in the prediction results. Thereafter, they combined it with neural networks to further improve the accuracy of the prediction; its application in the prediction of house prices in Hefei City achieved the expected results. Ge et al. (23) explained that artificial neural network (ANN) models are a method designed to automatically capture functional forms, allowing the discovery of hidden non-linear relationships between modeled variables. ANN models are ANN methods with the ability to map complex non-linear relationships between variables and have good predictive power. However, the “black box” nature of ANNs is a major limitation because they lack explanatory power. Wang et al. (24) used a delayed neural network model to forecast Singapore's social property prices; the model was successful in producing accurate fits and estimates. Li et al. (25) used hedonic price and neural network models to predict house prices, and compared the accuracy of the two models in predicting the impact of park green space accessibility on house prices. They likewise found that predictions of the neural network model are more accurate than that of the hedonic price model. Tables 1–3 present the specific explanatory research methods.

**Table 1:** Literature review of the hedonic price model.

**Title**	**References**	**Innovation**	**Conclusion**	**Suggestions**
A new approach to consumer theory	(13)	Regard the hedonic price model a well-established method based on consumer theory.	Quantifying the amount that home buyers are inclined to pony up for the green space and park land around their houses, decomposing the characteristic factors in real estate price fluctuations so that an analysis of the role of green area and parks on property prices can be achieved.	In this model, the research team extended it to the activity analysis of consumption theory and applied it to production theory.
Combining revealed and stated preference methods to value environmental amenities at residential locations	(14)	A synthesis of fairly recent stated reference approaches, discrete choice hedonic analysis, and proven revealed preference techniques.	Leveraging multiple strategies to analyze the Fairfield, connecticut housing market, which consists of a mixture of environmental amenities and whose coastal wetlands are improving in quality as a result of active restoration efforts.	These environmental benefit calculations are helpful to the Town of Fairfield in determining the scope and applicability of its restoration efforts. Based on these findings, the Pine Creek marsh and wetland complex's aesthetic benefits should increase after restoration. This assessment method will be helpful to other municipalities thinking about ecosystem restoration in urban and suburban settings.
The effects of urban green spaces on house prices in the upper northwest urban development area of Adana (Turkey)	(15)	The hedonic price model is employed to depict the affordability of greenfield real estate in the housing market and to compile systematic data that may have an impact on the physical planning decisions made for new urban development regions.	The greater price paid for a home with green space than for a home without green space is a blatant evidence of the market worth of green space, which is difficult to quantify.	Set up the conditions required to balance the use of housing resources and grow a market with a sustainable size.
Revealing preferences of Prague's homebuyers toward greenery amenities: The empirical evidence of distance-size effect	(16)	Discuss potential biases in analysis caused by measurement, context, and autocorrelation in space.	Residential property prices are anticipated to benefit significantly from the Special Protection Area's and the urban forest's close proximity.	Making choices about the best green supply becomes crucial because many of the benefits of green spaces are not produced through the market. In light of this, public bodies may find it useful to base their decisions on how urban vegetation affects residents' quality of life. This research team took into account the various vegetation types in a region that has recently been impacted by housing developments in order to further this theme.
A study on the determinants of apartment prices in busan–focusing on the East West	(17)	Using the hedonic price model and GIS technology, factors that influence the prices of apartment buildings primarily in the eastern and western parts of Busan were empirically analyzed.	The results show that there is a difference in flat prices between Busan's eastern and western regions, with the eastern region's prices being more influenced by parkland than the latter.	It is necessary to consider the factors influencing the price of flats, such as economic flats in the western regions where the price of flats is relatively low, in order to lessen the price gap between the east and west and for the sake of a balanced development between the regions. The elements affecting apartment prices must be actively taken into account when implementing a supply policy.
Research on the regional effect of public resources on housing price–An Empirical Study Based on the districts and counties of Shanghai	(18)	A theoretical analysis of the mechanisms and pathways by which public resources affect housing prices is followed by the development of an empirical model based on the tiebout model.	The effect of public resources on housing prices was found to be regionally stratified for all three categories, with the effect becoming stronger the closer one gets to urban areas.	Combining the research presented in this paper, some policy recommendations are made with the goal of achieving a balance between the quantitative and qualitative supply of public resources and actively implementing supply-side reforms for public resources that are appropriate for the local context. (1) Public service equalization based on population density should be actively promoted. (2) When it comes to the provision of public resources, the government should actively participate in market mechanisms. (3) Put supply-side reforms into place for public resources in accordance with regional circumstances.
The value of park and green space as reflected by house prices in Taiwan	(18)	The hedonic price model and GIS are used in the study to evaluate the ecological effects of parks and green areas in Kaohsiung City, Taiwan.	The best distance to raise housing prices is not within 500 meters of parks and other green areas.	People are more concerned with park availability and quantity, and distance to parks may not be a major factor.

**Table 2:** Literature review of the geographically weighted regression model.

**Title**	**References**	**Innovation**	**Conclusion**	**Suggestions**
A GWR-based study on spatial pattern and structural determinants of Shanghai's housing price	(20)	Using the average housing price data of 1,014 communities within the outer ring of Shanghai in December 2010, a geographically weighted regression model was constructed and compared with ordinary least squares (OLS) based model to reveal the spatial heterogeneity of housing prices in Shanghai communities and the influence of different influencing factors.	For each increase or decrease in the number of units, the influence of each factor on the price of the property is, in order of magnitude: time to completion, distance to CBD, greenery, park, metro station, supermarket and school.	The study concludes that geographically weighted regression, decomposed into local parameter estimates, which are superior to the global parameter estimates provided by OLS, it provides insight into the complex relationships between housing prices and spatially influential factors, and the visualization tool can present a more detailed picture of the overall urban housing price landscape in the form of maps, all of which are unmatched by traditional OLS.
Add to favorite get latest update study on the Harbin commercial housing prices based on geographical weighted regression	(21)	A model of commercial housing prices in Harbin based on spatially geographically weighted regression is given using residential-related data from 2014 to 2015 in each district of Harbin. The variables affecting the spatial variation of housing in Harbin are explored and analyzed.	The findings indicate that the primary variables influencing spatial differences in housing prices are greenery, schools, and hospitals. In contrast, the metro and parks have a less major impact on property expenses. The age of buildings and commercial areas can also raise housing costs.	Greenery coverage, distance from schools, hospitals have a significant role in raising the price of housing, and all contribute to the spatial variability in the price of commercial real estate in the city. To ensure that the development of the city is more balanced, organized, and harmonious, attention should be paid to these elements and the development of these infrastructures.
The impact of urban green space accessibility on house prices in Dalian City	(26)	This paper investigates the relationship between availability to urban green area and housing prices in Zhongshan District, Dalian City through proximate analysis, geographically weighted regression models and multiple sources of data (e.g., housing prices and green spaces).	With the exception of Guilin Street, which exhibits a localized peak in housing prices falling from the center of the street toward the periphery, there is a downward trend from the coast to the interior.	Several issues are brought up that merit investigation and have ramifications for future research. First, the cost of homes fluctuates over time. The data for this study was only gathered at one point in time, so it does not accurately reflect changes over time. Secondly, residential areas are represented by dots in this study. If the boundaries of each area had been recorded, the analysis would have been more precise. Thirdly, buildings were not taken into account when assigning the blank grid to the time cost values of other roads in order to calculate accessibility. Future research might include obstruction values for buildings to improve the accuracy of the accessibility calculations.

**Table 3:** Literature review of the neural network model.

**Title**	**References**	**Innovation**	**Conclusion**	**Suggestions**
Combination forecast model base on neural network and SVM -Its application of house prices forecast as an example	(22)	A combined prediction model is proposed–the error sum of the minimum standard absolute value of the linear combined model, which combines neural networks and support vector machines in multi-factor training with better advantages, further improving the accuracy of the prediction.	The anticipated outcomes are obtained when it is applied to the prediction of house prices in Hefei.	Simple and accurate neural networks are built to contribute to purely technical house price forecasts for the Chinese market. Results can be used on their own or combined with fundamental forecasts to form a view of house price trends and for policy analysis.
Forecasting Hong Kong housing prices: An artificial neural network approach	(23)	The study develops a method for predicting Hong Kong private residential property values using artificial neural network (ANN) techniques using quarterly aggregated economic indicators.	ANN techniques have good predictive power and can map intricate non-linear relationships between variables.	Neural network models have certain limitations. The model is learned and weights are adjusted during construction and there is no formula for actually expressing the model. In order to learn and produce the best outcomes, it also needs a lot of data. Further comparisons are therefore necessary.
Predicting public housing prices using delayed neural networks	(24)	A delayed neural network model was utilized in the study to forecast Singapore's public housing costs.	Delayed neural network models can produce good fits and predictions.	For calculating home prices, delayed neural network models are a useful resource. Despite this, it must still be used cautiously and with awareness of any potential flaws. This study's use of neural network models for price forecasting will be more rigorous in subsequent studies.
Measuring the impact of park accessibility on house prices based on neural network	(25)	An improved method based on the principles of the mean impact value (MIV) algorithm was used to ensure that the extent to which distance to the park affects house prices was quantified without creating outliers.	The price decreases with increasing distance from the park. The price of the neighborhood is most affected by the size of the parkland and the level of vegetation.	The use of an improved method based on the principles of the mean impact value (MIV) algorithm ensures that the degree of impact to park distance on house prices is quantified without outliers, broadening the application of neural network models in the quantification of the degree of impact of variables.

### Theme II: Overview of variability on the impact of the same or the same type of parkland on house prices

Heterogeneity of time, space and property should be considered when analyzing variables that affect house prices in parklands. As research advances, findings on the degree to which the same or the same type of parkland has a differential impact on house prices have become significant. Crompton (27) stated that the real estate value of parks adjacent to parks will have a positive impact of 20%. Zhang et al. (28) analyzed the impact of urban parklands on real estate values in Beijing by subdividing the parks into six categories by size and type to conduct a detailed analysis to strengthen the findings. They concluded that parklands can have a 0.5–14.1% value-added effect on property prices in the 850–160-m range in their vicinity. Tian (29) used the three free civic parks in Tianjin as examples and concluded that the impact of park green spaces on neighborhood property prices is less than that of traffic, and that the impact of parks on property prices varies between areas. Trojanek et al. (30) used the hedonic price approach, OLS, GLS and QR models to conclude that urban green space within 100 m of a home increases residential prices by 3–4%. Panduro et al. (31) used the hedonic pricing approach to explore the implicit price of urban green space availability. They concluded that within a 1,000-m radius park implicit price is an annual rent increase of 0.33% per annum (31). Evangelio et al. (32) provided a preliminary estimate of the impact of parks on house prices within Victoria. They estimated the hedonic regression of house prices in terms of distance to six types of parks and various other amenities that may affect house prices. Additionally, they determined that certain types of parks can have a significant positive impact on house prices. Liu and Chen (33) concluded that a 1-km decrease in distance to parklands is associated with an average increase in residential prices of RMB 21,840.

McCord et al. (34) concluded from early research on the hedonic price approach that parklands tend to have an appreciative impact on quality of life and property values. However, several research perspectives have emerged from previous studies. Some scholars have argued that different types of parks have an appreciating and depreciating impact on neighborhood property values. McMillan's (35) analysis of measuring the benefits generated by urban water parks was the beginning of research on the assessment of the economic benefits of urban parks on real estate. Subsequent studies in this area have mostly concluded that urban park green spaces have a positive impact on house prices. However, there is a subset of scholars with different findings, such as Pearson et al. (36), who studied the impact of Noosa National Park on the price of surrounding undeveloped land. They concluded that residential values increased by 7% with a small amount of additional green space resources. However, for properties located south of the park, the value was only 85% of that of properties in the north and did not generate an increase in value. Hobden et al. (37) conducted a statistical analysis of parks in suburban Canada. Particularly, they compared matched sales over a 20-year period and concluded that most types of green space increase the value of nearby single-family homes, with corridor green spaces having an impact on adjacent property values. However, there are some exceptions that negatively affect residential values. Nicholls and Crompton (38) selected three communities in the same city for comparison, two of which are adjacent to green spaces and produce significant property value appreciation benefits. Greenways were found to have a significant positive impact on the sales price of neighboring properties, but the reverse had a negative impact. Jim and Chen (39) purposefully compared the impact of community parks, streetscapes and oceanfront architectural views on surrounding house prices. They concluded that community parks add the most significant value to residential prices, followed by ocean views, boosting premiums by 5.1%, with streetscapes and architectural views being less attractive to residents, thereby resulting in depressing prices. Crompton and Nicholls (40) analyzed the literature on parks and development space on residential values outside North America by US scholars. They concluded that from 11 studies in China, five revealed a relationship of expected appreciation, four reported mixed results of appreciation and depreciation, and two showed no effect of parks on house prices. Three studies from Japan and Australia also showed different results, with subtle differences between the results. Tables 4, 5 provide specific explanatory variable details.

**Table 4:** Literature review of the variability on the impact of the same park green space.

**Title**	**References**	**Innovation**	**Conclusion**	**Suggestions**
The impact of parks on property values: Empirical evidence from the past two decades in the United States	(27)	Utilize the more sophisticated analytical techniques that social scientists now have access to review the current research.	The park increases the property values in the area by 20%.	The empirical results examined in this paper are significant because they offer acceptable estimates of the financial benefit for park proponents. Many senior officials' ruling models appear to be based on these metrics, and elected politicians who want to show “oversight” for public expenditure want them.
The effects of public green spaces on residential property value in Beijing	(28)	The effect of park distance on house prices and the added value coefficient was examined using the hedonic price model. Additionally, the effect of park green space distance on house values was investigated using two functional analysis methods. Finally, the context of growing of Beijing's green space in urban parks impacting real estate values was identified using GIS-assisted mapping.	The appreciation of a home is highly correlated with distance and declines with increasing distance.	The kind and shape of the parkland determines how much of an impact it has on the additional value of the nearby properties. This report is a crucial resource for Beijing and the rest of the nation's development of urban residential areas and management of green space resources.
A study on the impact of urban parks and green spaces on surrounding real estate prices in Tianjin	(29)	Using the three free municipal parks in Tianjin as an example, statistical analysis and hedonic pricing analysis are used to assess the geographical impact of urban parks on the real estate values of the nearby communities.	Parkland affects neighboring home prices less than transportation, and the effect of parks on home values varies by region.	The essential information for the joint development mechanism of real estate and green infrastructure is provided, as well as quantitative analysis tools and models for the study of housing economics and urban economics. This makes it easier for the government to create legislation, developers to plan development strategies, and consumers to make home purchases.
Effect of public green space on residential property values in Belfast metropolitan area	(34)	A large number of empirical studies have shown that public green spaces such as urban parks have a positive impact on property values. However, there are few empirical studies in this regard about the UK. This study aims to address this gap by examining the impact of public green space on house prices within a medium sized area in a UK city.	All else being equal, urban green spaces had a significant positive impact on the sales prices of neighboring residential properties in the terrace and condominium sector. In contrast, sales prices for terrace and condominium properties located closer to public green space increased by 49%. Of the four housing types analyzed, only two generated significant property value premiums due to their proximity to green open space.	With limited statistically significant direct impacts evident in the segregated and semi-segregated sectors, this finding has important social and public policy implications.
The effect of urban green spaces on house prices in Warsaw	(30)	The influence of near proximity to urban green space on Warsaw apartment prices was investigated using a hedonic price model together with OLS, GLS, and QR models.	Urban green space within 100 meters of a flat raises the cost of a home by 3.4–4.6%.	The latter findings could offer novel interpretations of earlier findings, particularly those by Trojanek (30) and Czembrowski and Kronenberg (41), which disregarded potential variations in urban green space hedonic costs for various types of residential communities. Both housing developers, who offer incentives for better project siting possibilities, and urban planners, who make cases for preserving and safeguarding parks and greenbelts in cities, may discover the findings to be of interest.
Eliciting preferences for urban parks	(31)	A method for imposing identification restrictions on utility functions is used to elicit preferences and gauge willingness to pay for functions for copenhagen park availability.	Within a 1,000-meter radius, the implied cost of the park is an increase of 53.25 euros per hectare per year. For the average flat, the annual rent increases by 0.33% per hectare.	The methodology put forward by Bajari and Benkard (42) is applied in this study to learn more about the preferences and ideals of urban parks. The functional form limitations of the utility function are used in this framework to identify preferences. This study also shows how urban parks have a non-negligible flow of value and how preferences for park accessibility alter with socio-demographic data, which may have intriguing policy implications. Thirdly, this study illustrates the significance of selecting these indicators correctly for the results of policy assessment through the use of two common park availability indicators.
What makes a locality attractive? Estimates of the amenity value of parks for Victoria	(32)	The impact of proximity to six distinct types of parks on Victoria home prices is estimated for the first time in this study. Additionally, distinct regressions are carried out for data from Melbourne and rural Victoria.	When a home is moved from the median to the first percentile of distance to the park, its value may rise by up to $86,000. In Victoria rather than Melbourne, parks are more likely to have a favorable effect on home prices. It stands to reason that various park types in Melbourne would experience traffic or other adverse externalities.	The findings of this study can be utilized to create quick cost-benefit analyses by estimating the worth of park amenities.
Impact of urban park green space on the price of peripheral housing in Urumqi	(33)	The study used Urumqi city as the study region and included 1,789 residential POI data from about 16 park green areas in six urban locations in 2019. From these data, 14 explanatory variables were chosen from the three dimensions of location, building structure, and neighborhood relationship. A residential market hedonic pricing model was then built and combined with elasticity and marginal price analysis to quantify the impact of park green areas on residential prices in Urumqi city.	In Urumqi, the distance to a park or other green space is inversely correlated with the cost of a residence.	It is conducive to enriching the research related to urban green space and house prices in Northwest China, and is of some significance in advancing the culture of urban green space externality evaluation as well as house price research.

**Table 5:** Literature review of differences in the direction of impact of the same park green space.

**Title**	**References**	**Innovation**	**Conclusion**	**Suggestions**
“Measuring Benefits Generated by Urban Water Parks”: Comment	(35)	Applying the idea of measuring the value of a commodity service to a study of the valuation of the benefits of parkland.	The value added increases with proximity to parkland.	It is intended that this note would aid in more precise estimation of the advantages (and costs) that the techniques employed by Darling and others have for evaluating amenities (and nuisance issues) in urban settings.
The impact of Noosa National Park on surrounding property values: An application of the hedonic price method	(36)	Hedonic pricing was used to determine how the Noosa National Park might affect the price of surrounding unimproved land.	It turns out that even a little increase in green space raises land values by 7%.	The method that works best for the kind of non-market valuation is probably the hedonic price approach. Future research should consider incorporating residential neighborhood values and landscapes in a GIS format to get a more precise and straightforward method of measuring environmental variables. It should be highlighted that this method can be used to value any environmental asset that affects the local population. Whether the region is a national park, a lake, a forested area, or another recreational area. To get precise forecasts from the research, a variety of elements influencing the land's value must be taken into consideration.
Green space borders–a tangible benefit? Evidence from four neighborhoods in Surrey, British Columbia, 1980–2001	(37)	Using a mean difference test, statistical analysis of matched sales pairings over 20 years in Canadian suburbs distinguishes specific forms of green space, with a focus on greenway corridors.	The value of neighboring single-family houses rises with most forms of green space, with corridors particularly having a large beneficial effect.	The study's findings add to the body of research showing how wise an investment parks and green areas are. Smaller green spaces, as opposed to normal parks, seem to be more valuable to single-family dwellings. The argument that “greenways can assist enhance property values as much as parks, or even more than huge parks” has some empirical support given that over half of the “small” park bridges have access. It is crucial that municipalities think through the viability of such corridors from a financial and policy standpoint.
The impact of greenways on property values: evidence from Austin, Texas	(38)	Investigate how greenways affect the value of the nearby residential properties using the hedonic price model.	The value of nearby houses might rise dramatically as a result of the Greenway. In two of the three areas, greenbelts close by offered substantial growth in home value.	In addition to the advantages the greenway offers in terms of the environment, society, aesthetics, and health, there are also the benefits in terms of recreation. The efficiency is high when viewed from an economic perspective. These amenities ought to be planned for from an urban perspective as a crucial component of a well-designed urban environment.
External effects of neighborhood parks and landscape elements on high-rise residential value	(39)	In order to quantify price differences caused by characteristics unique to certain commodities, statistical approaches were used to assess the external impact of community parks on the pricing of high-rise private residence in Hong Kong.	A community park has a 16.88% price increase potential.	Additional evaluations of the economic impacts of diverse urban landscapes in other locations, encompassing a wider variety of land uses and socioeconomic contexts, especially in the developing world, would be useful for upcoming investigations.
The impact on property values of distance to public parks and open spaces: findings from beyond North America	(40)	Studies from other non-North American countries were compared to the scope and characteristics of park influences on property values documented in American research to determine if the results corroborated or disagreed with those reported in other cultures.	The results vary among nations, but the general conclusion is that the distance between park and development space increases property values.	The second goal of this part is to discover methodological developments made by other cultural scholars that could improve the accuracy of calculations conducted in the US to estimate the influence of parks on real estate values. The emotional impact a park has on the neighborhood's population determines its quality, not just how it looks on the outside. The study's major concern is how to properly distribute recent findings so that they are appropriately taken into account in planning, social, and political decisions about community infrastructure.

### Theme III: Overview of the differential impact of different types of parkland on house prices

The extent to which different sizes, service radii and accessibility of parklands affect house prices relatively vary, and academic scholars are at a relatively advanced stage of research in this area. Some scholars have studied the extent to which different classes of parkland affect residential prices, concluding that the implicit value of different classes of parks on house prices varies. Panduro et al. (31) proposed a classification of green spaces into eight different types of spaces. They concluded that the types of green spaces with higher ratings in terms of accessibility and maintenance levels had higher implicit prices, whilst the types with lower ratings had lower implicit values. Wu et al. (43) analyzed the spatial distribution of green spaces and house prices at six scales in the central city of Suzhou. The distribution of park green spaces and real estate prices were significantly and positively correlated. Particularly, the scale of 2–10 hm^2^ park green space was closely related, and the clustering effect was prominent. Wu et al. (43) proposed an analysis of the impact of park green spaces at different scales, dividing parks into five classes according to size. The overall trend of “overlooked spot clustering–overlooked spot and hot spot clustering–hot spot clustering–divergence–insignificant” and the overall spatial distribution of park green spaces and property prices showed a significant positive correlation. The findings are an innovation on the basis of previous research findings and present new research ideas in this area. Subsequently, Wu and Shao (44) proposed a gradient difference in the correlation between parklands and housing prices at different service radii based on the analysis of parklands at different scales (with a distance gradient of 250 m). They concluded that housing with higher prices generally had more parklands around a 1,000-m service radius, and that for every 100-hm^2^ increase in parkland within 2,000 m of a residential area, housing unit prices increased by about RMB 1,000/m^2^. Many cases in the literature have shown that the accessibility of parklands is used as a differentiator for housing prices. Yang (45) focused on the choice of residential location and further investigated the effects of park size and distance on housing prices, concluding that houses will cost more the closer they are to urban parks and the bigger the parks' area are. Tan et al. (46) categorized accessibility issues from another perspective for comparison. The analysis of the impact on house prices in Fuzhou City delineated urban park accessibility within the Third Ring Road and urban park accessibility outside the Third Ring Road by proximity to the city center. They concluded that park accessibility within the Third Ring Road has higher impact on property prices than in urban areas outside the Third Ring Road, with higher accessibility leading to higher house prices overall. Liu (47) determined that the landscape accessibility factor has a negative relationship with residential prices, and that different landscapes accessibility factors have different effects on residential prices.

The degree of impact of different types of parkland on house prices in different areas can vary, and scholars in different countries have reached their respective conclusions. Kim et al. (48) selected flats in Busan, South Korea as subject of their study to categorize the temporal context of different plans for parks. Specifically, they integrated planned, implemented plans and completed parks to investigate what scale of park types were preferred by planners at different times. The degree of influence of parklands varies from one region to another and from one country to another. Chinese and foreign scholars have analyzed different types of parks and found that domestic residents prefer specialized types of parks with themes, whilst residents of some European and American countries prefer smaller community parks. Espey and Owusu-Edusei (49) found that proximity to small community parks had the greatest impact on house prices. Hobden et al. (37) analyzed four Surrey neighborhoods and found that most forms of green space increase the costs of single-family homes, with particular emphasis on the significant positive impact of ribbon greening on property values. Bark et al. (50) concluded a preference for green corridor-type parks in semi-arid urban areas. Pandit et al. (51) concluded that tree cover at the edge of a street is equivalent to a further 10% increase in other parks. However, Tan et al. (52) divided types of parks into five main categories and showed that strip parks have a smaller impact on house values and the greatest impact was seen in the dedicated park category. This difference is rooted in the different value demands that residents want parks and green spaces to provide. Chinese residents consider the location of strip parks and the limited thematic content they carry to be of a lower quality than specialized parks, and demand more content and quality from their parks. Residents in Europe and the US consider corridors and ribbon parks to be convenient for their daily exercise and considerably more accessible than large parks. They also consider that high-grade parks will inevitably create more noise and parking shortages, which will substantially reduce their home comfort. Therefore, they prefer to buy houses with small green belt parks nearby. Table 6 shows the specific explanatory variable details.

**Table 6:** Literature review of the impact of Parkland variability on housing prices.

**Title**	**References**	**Innovation**	**Conclusion**	**Suggestions**
Eliciting preferences for urban parks	(31)	A method for imposing identification restrictions on utility functions is used to elicit preferences and gauge willingness to pay for functions for copenhagen park availability.	Within a 1,000-meter radius, the implied cost of the park is an increase of 53.25 euros per hectare per year. For the average flat, the annual rent increases by 0.33% per hectare.	The methodology put forward by Bajari and Benkard (42) is applied in this study to learn more about the preferences and ideals of urban parks. The functional form limitations of the utility function are used in this framework to identify preferences. This study also shows how urban parks have a non-negligible flow of value and how preferences for park accessibility alter with socio-demographic data, which may have intriguing policy implications. Thirdly, this study illustrates the significance of selecting these indicators correctly for the results of policy assessment through the use of two common park availability indicators.
The relationship of spatial distribution between multi-scale park green space and housing prices: a case study of the Central City of Suzhou	(43)	The spatial distribution of green space and housing prices at six scales in Suzhou's center city were analyzed using the spatial autocorrelation approach.	Property prices and the distribution of park green space are significantly correlated positively. Particularly, the clustering impact and size of the 2- to 10-hm^2^ park green area are highly associated.	Understanding the spatial distribution of urban green spaces, their alterations, and their reactions to urban growth require a multi-scale viewpoint. It would be ideal if future studies looked at the social and ecological effects of various scales of urban green space designs.
Gradient difference of correlation between park green space and housing price in different service radius	(44)	The spatial analysis, correlation analysis and regression analysis were used to explore the relationship and gradients between the service radius of parkland from 500 to 2,000 m and the relationship between parkland and housing prices at different scales, using a distance gradient of 250 m.	As the scale of parkland increases, the overall correlation between service radius and housing price is “significant negative–highly significant positive–significant positive–not significant”.	(1) Seize the chance to increase the overall area of parkland by using the development of “park cities”. (2) Work to enhance the scale hierarchy of park green area. (3) Make park green spaces more effective in serving locals. (4) Make a stronger case for doing a thorough investigation into how parkland and urban features affect home prices.
A study on the influence of urban park location upon housing prices based on the Tiebout Choice Theory	(45)	A hedonic pricing model and a literature study were used to develop elements influencing home prices in order to quantify the influence and relevance of house prices on non-price environmental resources near urban parks. This helped to clarify residents' desire for urban parks.	The price increases with proximity to and size of the city park.	Optimizing the spatial organization of urban landscapes and building ecologically sustainable cities are both facilitated by the study of the effect of landscape on residential prices. It also helps to elucidate the mechanism by which urban residential price creation takes place.
The impact of accessibility of urban central parks on housing prices of Fuzhou	(46)	Based on remote sensing photography, web crawling, urban road network, and other sources, network analysis and SPSS correlation analysis are performed.	House prices and the distance to the park by foot are highly connected. The price increases as the distance is cut short.	Future urban planning insights and real estate market development ideas include: (1) Fuzhou Gaogai Mountain Park's entrances and exits should be thoughtfully planned, and more entrances and exits should be added, to increase security and accessibility. (2) To shorten the distance between dwellings and open places, the urban road network should be improved, and road building should be encouraged. This study provides a practical method for determining how the park affects the affordability of housing prices using ArcGIS shortest path, which can be used as a guide for the expansion of the real estate market and the development of an eco-livable city.
An analysis of the price of urban housing and the accessibility to ecological landscapes	(47)	It was discovered that the majority of prior research has concentrated on the effects of parkland and water bodies, two types of ecological landscape accessibility, on home prices. The price characteristics approach and the geographically weighted model are the two approaches most frequently used to study home prices.	Residential pricing and the landscape accessibility factor are negatively correlated, with different landscapes accessibility variables exerting varied pressures on residential costs.	More academics are focusing on landscape accessibility instead of natural landscapes, demonstrating how this factor affects residential pricing in a measurable and visual manner. But as this research is still in its early stages, more technological and theoretical development and refinement is still required.
Understanding the local impact of urban park plans and park typology on housing price: A case study of the Busan metropolitan region, Korea	(48)	The worth of future parks can also be assessed by looking into the correlation between property values and parks, just like it is done to estimate the value of existing parks. Three categories—“comprehensive plans”, “implementation plans” and “finished parks” are used in the analysis to describe the various planning settings for parks.	All already existing parks have a favorable effect on home prices, and smaller parks in populated neighborhoods were chosen throughout the park design process.	There is evidence to support the claim that parks raise urban environment enjoyment. A more precise assessment of the value generated by parks through urban parks and planning will be possible with further model development.
Neighborhood parks and residential property values in Greenville, South Carolina	(49)	To calculate the impact of park proximity on home values, a unique dataset of single-family homes sold in Greenville, South Carolina, between 1990 and 1999 was employed.	Small community parks close to residences have the most effects on property prices, and the benefits of these parks' proximity can be felt up to 1,500 feet away from the park.	Future developments of this study will focus on demographics and cross-cultural comparisons to determine how the geomorphic characteristics of various cities and towns, urban scale, and similarity to other forms of open area (for example, farms, land or state forests) affect the value of nearby parks.
Green space borders– a tangible benefit? Evidence from four neighborhoods in Surrey, British Columbia, 1980–2001	(37)	Using a mean difference test, statistical analysis of matched sales pairings over 20 years in Canadian suburbs distinguishes specific forms of green area, concentrating on greenway corridors.	Most forms of greenery boost the value of nearby single-family homes, with corridors in particular having a substantial favorable effect on the value of nearby properties.	The green corridor specifically promotes walking and bicycling, resulting in active spaces for the neighborhood and raising the value of residents' homes.
How do homebuyers value different types of green space?	(50)	Recognizing the trade-offs between preferences for constructed and natural green space in semi-arid urban locations.	Homebuyers in the study area prefer locations close to green spaces, notably community parks and riparian corridors, and proximity to green space can lead to higher home prices.	Given that the greenery of the riparian corridor is converted into residential values nearby, these public amenity green spaces provide positive benefits and the restoration of natural habitats may be an economic solution to rationalize water use.
The importance of tree cover and neighborhood parks in determining urban property values	(51)	An autocorrelated spatial least squares model was used to assess how neighborhood and environmental factors, including tree cover, affected the sales price of a single-family dwelling in Perth.	Tree cover at street edges (public spaces) and the extent of neighborhood parks and neighborhood green spaces have seen significant price growth in the Perth housing market.	Local governments should view the upkeep and expansion of parks and other protected spaces surrounding cities as a crucial component of their public policy since it benefits urban people on both a private and public level. In hedonic research, it is crucial to examine and account for spatial correlation (also known as spatial autocorrelation) in order to generate objective parameter estimates and, as a result, a marginal implied price that is more precise. The estimation of model parameters and marginal inferred prices is also influenced by the estimation method and the weight matrix chosen to describe the spatial link between data. Therefore, future research on spatial hedonic should contribute to the advancement of these modeling and estimation problems, including spatially dependent non-smooth models.
Effects of urban park green space accessibility on house price: a case study of central district of Fuzhou City	(52)	Using information from remote sensing photos, web crawling, the urban road network, and other sources, network analysis and SPSS correlation analysis were used to examine the effect of park accessibility on community property costs in Fuzhou's central city.	Parkland accessibility and home values are closely connected, with better accessibility resulting in higher home prices. Varying types of parkland have different association coefficients with property values: special parks > integrated parks > pleasure parks > strip parks > community parks. The accessibility of special parks has the greatest impact on house prices, while community parks have the least.	It is proposed to enhance the improvement of urban roads, increase the construction of tourist trails, and enhance the cooperation model between the government and real estate developers to jointly promote the development, construction and rational allocation of urban parks and green areas, forming a win-win mechanism.

### Theme IV: Retrospective overview on the perspective of diverse studies

Although the research on the impact of parklands on house prices has progressed, apart from the traditional direct research on the impact of parklands on changes in residential prices, the research has been suggested to possibly be transformed into studies on such aspects as capitalization issues and amenity issues of parklands. Kolbe and Wüstemann (53) showed that urban greenery, such as parklands, has a significant impact on the capitalization structure variable in real estate prices by calculating the coverage of parklands around dwellings and the continuous distance from them. Cai and Liu (54) proposed to translate the study into an assessment of the capitalization benefits of park green spaces. They showed that a 1% increase in distance to park green spaces is associated with a 0.020% decrease in residential unit prices. Bedell (55) investigated how to value the relationship with parks in terms of residential prices. Specifically, they proposed to capitalize on green spaces and water quality to assess the degree of impact on residential values. He concluded that proximity to parks has a negative impact on housing values, with a positive impact if there is greater continuity of park facilities valued. Amenity value of urban green spaces refers to the degree to which the ecological, economic and socio-cultural functions provided by these spaces are satisfying to urban residents. Yin et al. (56) referred to the concept of urban green space amenity and applied this implicit concept to a hedonic price model commonly used in economics to assess the impact of green space amenity on housing prices. They likewise provided advice to urban planners on the need for orderly “greening”. Qi (57) referred to the concept of amenity in her article titled “A Study on the Amenity of Parks and Green Spaces in Anyang City Based on Hedonic Price Method”, suggesting the necessity for urban planners and policy makers to have a more accurate grasp of the amenity of urban green spaces to effectively control and guide the expansion of urban spaces. Wen et al. (58) used Hangzhou as an example and used structural, neighborhood, location and landscape dimensions as explanatory variables. Additionally, they applied a residential hedonic price model to objectively assess the impact of various landscapes on house prices in terms of amenity. They concluded that when the proximity to West Lake and nearby parks increases by 1%, house prices decrease by 0.229 and 0.052%, respectively, whilst a 1% increase in park area increases house prices by 0.008%. Aliyu et al. (59) suggested that the presence of green spaces in parks enables residents to live in a more enjoyable environment and substantially increases personal wellbeing. Extensive surveys have indicated that parks positively affect property values, with planned parks and recreational open space community parks adding the most value. Research on parkland amenity issues has confirmed the reliability of the hedonic price approach in existing parkland studies and its potential application to planned parklands (60). Sohn et al. (61) evaluated the market value of residential properties in four zoning districts in Houston and concluded that living near green spaces has a positive capitalizing effect on the costs of a single-family property. Accordingly, living nearby promotes house price increases over a 10-year period. Table 7 shows the context of diverse studies.

**Table 7:** Literature review on the context of diverse studies.

**Title**	**References**	**Innovation**	**Conclusion**	**Suggestions**
Estimating the value of urban green space: a hedonic pricing analysis of the housing market in Cologne, Germany	(53)	Cross-geocoded data for several types of urban green space (UGS), such as parks and woods, were included to analyze the capitalization of UGS in real estate values, and geocoded data for water bodies and fallow land were also included to control for other open space categories.	UGS like parks and forests have a greater effect on capitalized structural factors in real estate values, with parks having a positive price effect.	Urban planners need to take into account urban green spaces. UGS is a valuable resource for urban dwellers in addition to its ecological advantages. Future research could employ additional geocoded data on the quality of open space to provide more in-depth analysis of the economic value of urban green spaces for planning and management reasons.
The capitalized effect of park green space on residential housing prices in central District of Changchun	(54)	A residential hedonic price model was constructed to quantitatively assess the direction and extent of the capitalization effect of park green space in Changchun.	In Changchun, there is a negative relationship between the price of a residential unit and the distance to a park, with a 1% increase in the proximity to a park leading to a 0.020% drop in the price of a residential unit.	To assess the effect of school quality on residential price appreciation, additional scientific quantification of residential attributes is required. Increase the depth of the analysis of how parkland evolves over time, for instance by gathering information on housing and green space across a number of years to examine how distance and value-added impacts alter.
Capitalization of green space and water quality into residential housing values	(55)	Examined the best way to gauge how close a residence is near parks, historic areas, and designated bodies of water in terms of how much it will cost to live there.	The impact of parkland on house prices is mixed.	In order to identify negative effects, future research will need to evaluate both the general public and the housing populations residing close to the park. The benefit of utilizing park amenities to address engineering fixes for park issues.
Impact of the amenity value of urban green space on the price of house in Shanghai.	(56)	Based on the GIS spatial analysis function, the hedonic price model was used to select four categories of housing structure, accessibility, area and landscape index with a total of 23 factors to quantitatively analyze the influence of major urban green area types on dwelling costs in Shanghai.	Urban green areas provide valuable amenities, and their location and kind of area have a substantial influence on home values, however the extent of this impact varies, e.g., park green accessibility has the greatest impact, while square green accessibility has a smaller impact.	(1) The number of green spaces should be increased in urban centers by principles of “greenery where possible” and increasing the concentration of green spaces. (2) In the planning of urban green spaces, attention should be paid to the concept of “people-oriented”, so that the layout of urban green spaces can be integrated with different classes and groups as far as possible.
A Study on the Amenity of Parks and Green Spaces in Anyang City Based on Hedonic Price Method	(57)	In the analyzing the amenities of existing parkland using the hedonic pricing technique, the method was also applied exploratorily to the study of the potential amenity of planned parkland, evaluating the amenity of existing parkland and the potential amenity of planned parkland, respectively. This allowed for the identification of issues and the formulation of reasonable recommendations.	The amenity of a park improves with size, whereas the amenity of an open green area does not increase with size. Besides, the size of the park has a much less effect on housing costs than the distance to it.	City management should be careful to improve the upkeep of current parks in the future to continue maximizing their amenity. In order to maximize the potential amenity of planned parks, existing plans should be adhered to during construction. Care should be taken to balance the influence of size and its distribution on amenity so that the new parks can demonstrate good amenity.
Assessing amenity effects of urban landscapes on housing price in Hangzhou, China	(58)	In order to statistically assess the influence of varied landscapes on the comfort of home prices utilizing four dimensions of explanatory variables-structure, neighborhood, location, and landscape-a residential hedonic price model was constructed using Hangzhou as an example.	The inner city of Hangzhou is home to a wide variety of landscapes, including mountains, lakes, rivers, and parks. There are several urban water features and green areas, and the surroundings are breathtaking. In order to statistically assess the implied values of various landscapes, this study gathers information on housing features, such as home prices, from six of Hangzhou's major metropolitan regions, and develops a housing hedonic price model.	The enhancement of urban quality of life is significantly influenced by urban landscape. The quantitative analysis of how the urban environment affects home values has real-world implications and can give relevant government agencies a theoretical foundation on which to develop public policy.
Do neighborhood parks and open space influence the values of surrounding housing prices? A research review and synthesis	(59)	Proposing that most studies rely on a narrow definition of open space and that the conclusions lack generalisability, the study broadens the scope of the review.	Property values in communities with planned parks and recreational open space will be greater than in those without such facilities.	A full model should be developed in the future with greater functionality and empirical data from a large number of real transactions. The study's findings represent an essential first step in evaluating the overall advantages of keeping open space in urban settings.
Impact of lake landscape on urban residential property values in Nanjing	(60)	The famous lake view Mochou Lake in Nanjing was chosen as the sample. The Hedonic residential price model P = f(L,S,N) was applied to quantify the effect of a particular lake view on residential prices.	Mochou Lake has a strong favorable effect on the price of nearby residential properties, with the lake's comfort value or ecological services accounting for around 13% of those prices.	(1) Planning for both urban development and land use should take into account the impact of landscape variables. (2) The effect of landscape on residential prices should be taken into account in the process of property taxation. (3) The value of the landscape should be incorporated into the city's land and property price assessment system. (4) The value of landscape should be fully considered in the product development and pricing of real estate developers.
The capitalized amenity of green infrastructure in single-family housing values: An application of the spatial hedonic pricing method	(61)	The study created cross-sectional and longitudinal house pricing models as well as traditional hedonic price models and spatial econometric models to calculate the capitalization impact of parks.	According to the study's results, converting drainage infrastructure into amenities like parks is crucial for raising the community's aesthetic value and bringing about long-term economic advantages.	In order to provide long-term value for the community as an industry for the environment, society, and economy, stormwater treatment ponds are more methodically and properly planned.

## Conclusion

Research on the impact of urban parklands on house prices has been well-referenced, and this paper provides a categorized review of previous studies in terms of research methods, findings and research perspectives. From a research perspective, previous research methods have focused on the common hedonic price modeling, GWR and neural network modeling approaches, all of which have been relatively well-established and remain widely used in relevant price system studies. The review of the findings is divided into two main sections: (1) differential impact of the same type of parkland on the price of different homes and (2) differential impact of differential parkland on the price of homes. The final section reviews other relevant research perspectives on the impact of parklands on house prices. We are optimistic that this literature review will be useful for future scholars to engage in related research directions at the supplementary and reference levels. We are likewise looking forward to scholars and researchers supplementing and improving previous studies in terms of more research methods, research subjects and research perspectives, so that research on the impact of parklands on house prices can be more mature to serve all people with relevant opinions.

Lastly, there are also some possible future research directions suggested in this article. Studies are limited on how urban parklands affect pricing when other factors are considered, even though research on the impact of parks on housing prices is relatively well-established. Additionally, there is a dearth of study on the variations in the effect of parkland variables on home prices over time, and the majority of previous studies have focused on the effects of parkland variables on house prices at the same period. Additionally, earlier research has concentrated more on the association between parklands' effects on home values and those prices than on the cause of that effect. In the future, the causality of parks on home prices should be explored using more comparative methodologies, such as the difference-in-differences model.

## Author contributions

KC contributed to conception and design of the study. HL organized the database. KC and SY performed the statistical analysis. KC and HL wrote the first draft of the manuscript. SY and YH wrote sections of the manuscript. All authors contributed to manuscript revision, read, and approved the submitted version.

## Funding

This study was supported by a grant from Chengdu Park City Demonstration Zone Construction Center (Chengdu gongyuan chengshi shifan qu jianshe yanjiu zhongxin) (No. GYCS2021-YB004).

## Conflict of interest

The authors declare that the research was conducted in the absence of any commercial or financial relationships that could be construed as a potential conflict of interest.

## Publisher's note

All claims expressed in this article are solely those of the authors and do not necessarily represent those of their affiliated organizations, or those of the publisher, the editors and the reviewers. Any product that may be evaluated in this article, or claim that may be made by its manufacturer, is not guaranteed or endorsed by the publisher.

## References

[1] Yang L Tang X Yang H Meng F Liu J. Using a system of equations to assess the determinants of the walking behavior of older adults. Transactions in GIS. (2022) 26:1339–54. 10.1111/tgis.12916

[2] Yang L Ao Y Ke J Lu Y Liang Y. To walk or not to walk? Examining non-linear effects of streetscape greenery on walking propensity of older adults. J Transp Geogr. (2021) 94:103099. 10.1016/j.jtrangeo.2021.103099

[3] Yang L Liu J Liang Y Lu Y Yang H. Spatially varying effects of street greenery on walking time of older adults. ISPRS Int J Geo Inf. (2021) 10:596. 10.3390/ijgi10090596

[4] Wimalasiri N Hemakumara G. GIS Based Analysis To Assess The Benefits Of Green Areas In The Urban Core (With Special Reference To The Vihara Maha Devi Park In Colombo. Bangalore: IJRET (2016).

[5] Sadeghian MM. The Health Benefits of Urban Green Spaces, a Literature Review. New Delhi: IJARIIE (2018).

[6] Banzhaf HS Farooque O. Interjurisdictional housing prices and spatial amenities: Which measures of housing prices reflect local public goods? Reg Sci Urban Econ. (2013) 43:635–48. 10.1016/j.regsciurbeco.2013.03.006

[7] Yang L Zhou J Shyr OF Huo DD. Does bus accessibility affect property prices? Cities. (2019) 84:56–65. 10.1016/j.cities.2018.07.005

[8] Yang L Liang Y He B Lu Y Gou Z. COVID-19 effects on property markets: the pandemic decreases the implicit price of metro accessibility. Tunnell Underground Space Technol. (2022) 125:104528. 10.1016/j.tust.2022.104528

[9] Hagerty JK Stevens TH Allen PG More T. Benefits from urban open space and recreational parks: a case study. J Northeast Agric Econ Council. (1982) 11:13–20. 10.1017/S0163548400003125

[10] Crompton JL. The Proximate Principle: The Impact of Parks, Open Space and Water Features on Residential Property Values and the Property Tax Base. Ashburn: National Recreation and Park Association (2004).

[11] Yang L Chau KW Szeto WY Cui X Wang X. Accessibility to transit, by transit, and property prices: Spatially varying relationships. Transport Res Part D Transp Environ. (2020) 85:102387. 10.1016/j.trd.2020.102387

[12] Yang L Chu X Gou Z Yang H Lu Y Huang W. Accessibility and proximity effects of bus rapid transit on housing prices: heterogeneity across price quantiles and space. J Transp Geogr. (2020) 88:102850. 10.1016/j.jtrangeo.2020.102850

[13] Lancaster KJ. A new approach to consumer theory. J Polit Econ. (1966) 74:132–57. 10.1086/259131

[14] Earnhart D. Combining revealed and stated preference methods to value environmental amenities at residential locations. Land Econ. (2001) 77:12–29. 10.2307/3146977

[15] Altunkasa MF Uslu C. The effects of urban green spaces on house prices in the upper northwest urban development area of Adana (Turkey). Turk J Agric Forest. (2004) 28:203–9. 10.13140/RG.2.2.28625.43367

[16] Melichar J Kaprová K. Revealing preferences of Prague's homebuyers toward greenery amenities: the empirical evidence of distance-size effect. Landsc Urban Plann. (2013) 109:56–66. 10.1016/j.landurbplan.2012.09.003

[17] Lee O Chol J. A study on the determinants of apartment prices in busan—focusing on the east west. Resident Environ Institut Korea. (2015) 13:53–66. Available online at: https://www.dbpia.co.kr/journal/articleDetail?nodeId=NODE06386695&language=ko_KR&hasTopBanner=true

[18] Chen X. Research on the Regional Effect of Public Resources on Housing Price–An Empirical Study Based on the districts and counties of Shanghai (Master's Thesis). East China Normal University, Shanghai (In Chinese) (2016).

[19] Lai P. The Value of Park and Green Space as Reflected by House Prices in Taiwan (No. eres2017_353). Delft: European Real Estate Society (ERES) (2017).

[20] Tang Q Xu W Ai F. A GWR-based study on spatial pattern and structural determinants of Shanghai's housing price. Econ. Geogr. (2012) 32:7 (in Chinese) 10.1109/GeoInformatics.2011.598072327295638

[21] Zhang B Cao L Wang L Gao Y. Add to favorite get latest update study on the harbin commercial housing prices based on geographical weighted regression. Nat Sci J Harbin Normal Univ. (2016) 3:11–14 (in Chinese). 10.3969/j.issn.1000-5617.2016.03.004

[22] Xu F Xie R Hao J. Combination forecast model base on neural network and SVM–Its application of house prices forecast as an example. J Chaohu College. (2012) 6:1–7 (in Chinese). 10.3969/j.issn.1672-2868.2012.06.003

[23] Ge JX Runeson G Lam KC. Forecasting Hong Kong housing prices: an artificial neural network approach. In: International Conference on Methodologies in Housing Research. Stockholm (2003).

[24] Wang L Chan F Wang Y Chang Q. Predicting public housing prices using delayed neural networks. In: 2016 IEEE Region 10 Conference (TENCON). Singapore: IEEE (2016). p. 3589–92.

[25] Li W Niu Z Zhang Y. Measuring the impact of park accessibility on house prices based on neural network. Price Monthly. (2020) 9:7–13 (in Chinese). 10.14076/j.issn.1006-2025.2020.09.02

[26] Yang J Bao Y Jin C. The impact of urban green space accessibility on house prices in dalian city. Scientia Geographica Sinica. (2018) 38:1952–60. 10.13249/j.cnki.sgs.2018.12.002

[27] Crompton JL. The impact of parks on property values: empirical evidence from the past two decades in the United States. Manag Leisure. (2005) 10:203–18. 10.1080/13606710500348060

[28] Zhang B Xie G Xia B Zhang C. The effects of public green spaces on residential property value in Beijing. J Resour Ecol. (2012) 3:243–52. 10.5814/j.issn.1674-764x.2012.03.007

[29] Tian Y. A study on the impact of urban parks and green spaces on surrounding real estate prices in Tianjin. J Southeast Univ. (2013) 92–7 (in Chinese). 10.13916/j.cnki.issn1671-511x.2013.s2.032

[30] Trojanek R. The impact of green areas on dwelling prices: the case of Poznań city. Entrepreneur Bus Econom Rev. (2016) 4:27–35. 10.15678/EBER.2016.040203

[31] Panduro TE Jensen CU Lundhede TH von Graevenitz K Thorsen BJ. Eliciting preferences for urban parks. Reg Sci Urban Econ. (2018) 73:127–42. 10.1016/j.regsciurbeco.2018.09.001

[32] Evangelio R Hone S Lee M Prentice D. What makes a locality attractive? Estimates of the amenity value of parks for Victoria. Econ Papers. (2019) 38:182–92. 10.1111/1759-3441.12259

[33] Liu Y Chen T. Impact of urban park green space on the price of peripheral housing in Urumqi. J Arid Land Resour Environ. (2020) 11:36–43 (in Chinese). 10.13448/j.cnki.jalre.2020.295

[34] McCord J McCord M McCluskey W Davis PT McIlhatton D Haran M. Effect of public green space on residential property values in Belfast metropolitan area. J Finan Manag Prop Constr. (2014) 19:117–37. 10.1108/JFMPC-04-2013-0008

[35] McMillan M. “Measuring Benefits Generated by Urban Water Parks”: Comment. Land Econ. (1975) 51:379–81. 10.2307/3144955

[36] Pearson LJ Tisdell C Lisle AT. The impact of Noosa National Park on surrounding property values: an application of the hedonic price method. Econ Anal Policy. (2002) 32:155–71. 10.1016/S0313-5926(02)50023-0

[37] Hobden DW Laughton GE Morgan KE. Green space borders–a tangible benefit? Evidence from four neighborhoods in Surrey, British Columbia, 1980–2001. Land Use Policy. (2004) 21:129–38. 10.1016/j.landusepol.2003.10.002

[38] Nicholls S Crompton JL. The impact of greenways on property values: evidence from Austin, Texas. J Leis Res. (2005) 37:321–41. 10.1080/00222216.2005.11950056

[39] Jim C Chen W. External effects of neighbourhood parks and landscape elements on high-rise residential value. Land Use Policy. (2010) 27:662–70. 10.1016/j.landusepol.2009.08.027

[40] Crompton J Nicholls S. The impact on property values of distance to public parks and open spaces: findings from beyond North America. World Leis J. (2022) 64:61–78. 10.1080/16078055.2021.1910557

[41] Czembrowski P Kronenberg J. Hedonic pricing and different urban green space types and sizes: insights into the discussion on valuing ecosystem services. Landsc Urban Plann. (2016) 146:11–19. 10.1016/j.landurbplan.2015.10.005

[42] Bajari P Benkard CL. Demand estimation with heterogeneous consumers and unobserved product characteristics: a hedonic approach. J Polit Econ. (2005) 113:1239–76. 10.1086/498586

[43] Wu D Shao D Liu Z Yu H Wang J. The relationship of spatial distribution between multi-scale park green space and housing prices: a case study of the Central City of Suzhou. Chin Landsc Architect. (2018) 11:113–8 (in Chinese). 10.3969/j.issn.1000-6664.2018.11.023

[44] Wu D Shao D. Gradient difference of correlation between park green space and housing price in different service radius. J China Urban Forest. (2020) 4:50–4 (in Chinese). 10.12169/zgcsly.2019.04.25.0001

[45] Yang S. A Study on the Influence of Urban Park Location upon Housing Prices based on the Tiebout Choice Theory (Master's Thesis). Ming Chuan University, Taoyuan, Taiwan (2007).

[46] Tan W Liu L Cui Y Chen J Lin F Zhong Y. The impact of accessibility of urban central parks on housing prices of Fuzhou. In: E3S Web of Conferences. Vol. 165. Changchun: EDP Sciences (2020).

[47] Liu Q. Research on the impact of urban landscape accessibility on housing prices in the central area of Chongqing (Master's Thesis). Southwest University, Chongqing, China. (2021). 10.27684/d.cnki.gxndx.2021.002903

[48] Kim HS Lee GE Lee JS Choi Y. Understanding the local impact of urban park plans and park typology on housing price: a case study of the Busan metropolitan region, Korea. Landsc Urban Plan. (2019) 184:1–11. 10.1016/j.landurbplan.2018.12.007

[49] Espey M Owusu-Edusei K. Neighborhood parks and residential property values in Greenville, South Carolina. J Agric Appl Econ. (2001) 33:487–92. 10.1017/S1074070800020952

[50] Bark RH Osgood DE Colby BG Halper EB. How do homebuyers value different types of green space? J Agric Resour Econ. (2011) 395−415. 10.22004/ag.econ.117210

[51] Pandit R Polyakov M Sadler R. The importance of tree cover and neighbourhood parks in determining urban property values.In: 56th AARES Annual Conference. Fermantle, WA: Australian Agricultural and Resource Economics Society (2012).

[52] Tan W Chen T Wang Q Lin F Huang Y Cui Y. Effects of urban park green space accessibility on house price:a case study of central district of Fuzhou City. J China Urban Forest. (2021) 1:66–71 (in Chinese).

[53] Kolbe J Wüstemann H. Estimating the value of urban green space: a hedonic pricing analysis of the housing market in cologne, Germany. Folia Oecon Stet. (2014). p. 43–58. 10.13140/2.1.3777.1048

[54] Cai W Liu Z. The capitalized effect of park green space on residential housing prices in central district of Changchun. Hubei Agric Sci. (2017) 14:2768–72 (in Chinese). 10.14088/j.cnki.issn0439-8114.2017.14.043

[55] Bedell WB. Capitalization of Green Space and Water Quality Into Residential Housing Values (Master Thesis). University of Kentucky, Kentucky, United States (2018).

[56] Yin H Xu J Kong F. Impact of the amenity value of urban green space on the price of house in Shanghai. Acta Ecol Sin. (2009) 29:4492–500 (in Chinese). 10.3321/j.issn:1000-0933.2009.08.057

[57] Qi J. A Study on the Amenity of Parks and Green Spaces in Anyang City Based on Hedonic Price Method (Master's Thesis). Sichuan Agricultural University, Ya'an (in Chinese) (2013).

[58] Wen H Zhang Y Zhang L. Assessing amenity effects of urban landscapes on housing price in Hangzhou, China. Urban Forest Urban Greeni. (2015) 14:1017–26. 10.1016/j.ufug.2015.09.013

[59] Aliyu AA Adamu H Gambo MJ Auwal U Raymond D. Do neighbourhood parks and open space influence the values of surrounding housing Prices? A research review and synthesis. In: Book of Proceedings/Abstract and Programme: the 10th Academic Conference of Hummingbird Publications and Research International on Challenge and Prospects Vol.11 No.1 on 4th August, 2016-Conference Hall, Administrative Complex, Osun State University. Oso (2016).

[60] Wu D Guo Z Chen H. Impact of lake landscape on urban residential property values in Nanjing. Resour Sci. (2008) 30:1503–9 (in Chinese).

[61] Sohn W Kim HW Kim JH Li MH. The capitalized amenity of green infrastructure in single-family housing values: an application of the spatial hedonic pricing method. Urban Forest Urban Green. (2020) 49:126643. 10.1016/j.ufug.2020.126643

